# A cross‐sectional study evaluating the frequency of HIV drug resistance mutations among individuals diagnosed with HIV‐1 in tenofovir disoproxil fumarate‐based pre‐exposure prophylaxis rollout programmes in Kenya, Zimbabwe, Eswatini and South Africa

**DOI:** 10.1002/jia2.70011

**Published:** 2025-08-20

**Authors:** Urvi M. Parikh, Lauren D. Kudrick, Lisa Levy, Everline Bosek, Bhavna H. Chohan, Irene Mukui, Sarah Masyuko, Nonhlanhla Ndlovu, Imelda Mahaka, Owen Mugurungi, Gertrude Ncube, Anita Hettema, Sindy N. Matse, Saiqa Mullick, Carole L. Wallis, Amy L. Heaps, Kerri J. Penrose, Kevin D. McCormick, Lubbe Wiesner, Peter L. Anderson, Jill M. Peterson, Connie Celum, Barbra A. Richardson, Delivette Castor, Shannon Allen, Kristine Torjesen, John W. Mellors, Global Evaluation of Microbicide Sensitivity (GEMS) Project

**Affiliations:** ^1^ University of Pittsburgh Pittsburgh Pennsylvania USA; ^2^ FHI 360 Durham North Carolina USA; ^3^ University of Washington‐Kenya Nairobi Kenya; ^4^ Kenya Medical Research Institute (KEMRI) Nairobi Kenya; ^5^ Departments of Global Health Medicine, Epidemiology and Biostatistics University of Washington Seattle Washington USA; ^6^ National AIDS and STI Programme Ministry of Health Nairobi Kenya; ^7^ Pangaea Zimbabwe Harare Zimbabwe; ^8^ Ministry of Health and Child Care Harare Zimbabwe; ^9^ Clinton Health Access Initiative Mbabane Eswatini; ^10^ Ministry of Health Mbabane Eswatini; ^11^ Wits RHI The University of the Witwatersrand Johannesburg South Africa; ^12^ BARC‐SA and Lancet Laboratories Johannesburg South Africa; ^13^ Division of Clinical Pharmacology Department of Medicine University of Cape Town Cape Town South Africa; ^14^ University of Colorado‐Anschutz Medical Campus Aurora Colorado USA; ^15^ Columbia University Irving Medical Center New York New York USA; ^16^ United States Agency for International Development (USAID) Washington DC USA

**Keywords:** HIV, HIV prevention, pre‐exposure prophylaxis, drug resistance, tenofovir disoproxil fumarate/emtricitabine, sub‐Saharan Africa

## Abstract

**Introduction:**

The ongoing rollout of oral tenofovir‐based pre‐exposure prophylaxis (PrEP) has the potential to reduce HIV‐1 incidence, but HIV drug resistance (HIVDR) in individuals who acquire HIV‐1 on PrEP could threaten the treatment effectiveness of overlapping antiretrovirals (tenofovir/emtricitabine), contribute to development of resistance, and undermine HIV control efforts. Accordingly, the Global Evaluation of Microbicide Sensitivity (GEMS) project was established to monitor HIVDR in PrEP rollout programmes in Southern and Eastern Africa.

**Methods:**

GEMS monitored resistance in >100,000 estimated persons who accessed PrEP through national programmes or implementation projects in Southern/Eastern Africa. Participants self‐reported demographics and PrEP adherence. HIV‐1 RNA and tenofovir‐diphosphate levels were measured in blood samples collected at the time of study enrolment from consenting participants diagnosed with HIV who had received PrEP. HIVDR mutations were detected by population genotyping.

**Results:**

Of 283 reported seroconversions on PrEP from December 2017 through September 2023, 255 (90%) individuals enrolled in GEMS, of which 81 (32%) were from Kenya, 77 (30%) from South Africa, 69 (27%) from Zimbabwe and 28 (11%) from Eswatini. Half (130; 51%) were 15–24 years of age at seroconversion, and three‐quarters (193; 76%) were female. Thirty‐four seroconversions occurred within 30 days of PrEP initiation. Tenofovir‐diphosphate levels were consistent with moderate to high levels (≥350 femtomoles per punch) in 53% (120 of 226) individuals with drug‐level data. Of 154 samples successfully genotyped, 34 (22%; 95% CI [16%, 30%]) had PrEP‐associated mutations; these included 27 samples with M184I/V, one sample with K65KR, and six samples with both K65R and M184I/V.

**Conclusions:**

The frequency of HIVDR mutations associated with tenofovir or emtricitabine among individuals diagnosed with HIV who had received PrEP (22%) exceeded background levels of transmitted nucleoside *reverse transcriptase* inhibitor resistance in Southern and Eastern Africa (≤5%) but people with PrEP‐associated mutations are likely to achieve virologic suppression with current first‐line antiretroviral therapy (ART). Improved screening for acute infection before initiating PrEP, surveillance of HIVDR with the introduction of new PrEP programmes and the monitoring of longer‐term ART outcomes in individuals who acquire HIV‐1 on PrEP will be essential to preserve antiretroviral options for both treatment and prevention.

## 1 INTRODUCTION

Nearly a decade ago, the World Health Organization (WHO) recommended daily oral tenofovir disoproxil fumarate (TDF) with either emtricitabine or lamivudine (abbreviated XTC) pre‐exposure prophylaxis (PrEP) as part of a combination HIV prevention [1]. As of the first quarter of 2024, over 6.7 million individuals had initiated oral PrEP with TDF/XTC. Kenya, Zimbabwe, Eswatini and South Africa were among the first African countries to approve TDF/XTC nationally, deliver PrEP through routine public service and scale up PrEP initiation; more than half of PrEP initiations have occurred in Southern and Eastern Africa [2, 3, 4, 5]. Most countries in Africa follow WHO recommendations for HIV testing strategies with PrEP, which include following national standard‐of‐care testing algorithms (e.g. non‐reactive antibody‐based rapid diagnostic test) or self‐testing prior to starting oral PrEP, with recommended follow‐up testing in 1 month, then repeat testing every 3 months thereafter while continuing oral PrEP [6]. PrEP coverage has been associated with a decrease in new HIV‐1 diagnoses in high‐income settings such as the United States and Australia. PrEP has demonstrated success in reducing HIV‐1 incidence in key populations, but protection decreases with inconsistent PrEP access and persistence [7, 8, 9, 10, 11, 12].

TDF and XTC remain integral components of current first‐line antiretroviral therapy (ART) in many countries, thus raising the concern that individuals who acquire HIV‐1 while on PrEP could select for drug resistance that subsequently decreases the effectiveness of future ART. In the initial Phase III clinical trials of PrEP, the frequency of TDF‐ or FTC‐associated resistance was higher (41%) in individuals who started PrEP during the undetected acute stage of HIV‐1 acquisition compared to individuals who seroconverted later in the study and were retrospectively confirmed to be HIV‐1 negative at enrolment (3%) [13, 14].

A published pooled analysis of 72 global post‐approval implementation studies and demonstration projects found that 22 of 78 (28%) seroconversions on PrEP had genotypic resistance, with the following HIV‐1 *reverse transcriptase* mutations: TDF‐associated K65R (one case), XTC‐associated M184I/V (18 cases), or both (three cases) [15]. Individuals with high or variable PrEP adherence have also been reported to have K65R and/or M184I/V mutations detected at HIV‐1 diagnosis [16, 17, 18, 19, 20]. The frequency of PrEP‐associated resistance from cohorts within clinical settings ranged from 5% to 44% [21, 22, 23, 24, 25, 26, 27]. By contrast, rates of transmitted nucleoside reverse transcriptase inhibitor (NRTI) resistance remain below 5% in Kenya and South Africa and 6.1% overall in Africa [28, 29, 30, 31, 32, 33].

WHO has highlighted the importance of monitoring for HIV‐1 drug resistance (HIVDR) in the context of PrEP in national programmes, recommending cross‐sectional, time‐limited, passive surveillance of PrEP initiators [34]. Here, we report the frequency of HIVDR in PrEP users participating in national PrEP programmes or delivery projects in Kenya, South Africa, Zimbabwe and Eswatini who were diagnosed with HIV‐1.

## 2 METHODS

### 2.1 Study design

The Global Evaluation of Microbicide Sensitivity (GEMS) project, a cross‐sectional, observational study, assessed the frequency of HIVDR in individuals diagnosed with HIV‐1 while on oral TDF/FTC or TDF/lamivudine (3TC) PrEP, from December 2017 through September 2023 across four countries: Kenya, Zimbabwe, Eswatini and South Africa. All protocols (except in South Africa) were nationally led and conducted in collaboration with each country's Ministry of Health and local non‐governmental organizations.

In Kenya, the study was approved by the Kenya Medical Research Institute Scientific and Ethics Review Unit through the Center for Virus Research on 5 March 2018 (KEMRI/SERU/CVR/018/ 3636). In Eswatini, the study was approved by the Eswatini Health and Human Research Review Board on 23 January 2020 (Board Registration Number FWA 00026661/IRV 00011253; Protocol Number SHR199/2019). In Zimbabwe, the study was approved by the Medical Research Council of Zimbabwe on 6 September 2018 (MRCZ/A/2309). In South Africa, PrEP was offered programmatically to female sex workers and transgender clients at limited sites through the Wits RHI Key Populations Programme; resistance monitoring for seroconversions was conducted through an independent protocol approved by the Human Research Ethics Committee (Medical) at the University of the Witwatersrand, Johannesburg, on 17 July 2019 (Reference R14/49; Protocol Number M190542).

In all other partner projects in South Africa and Kenya, PrEP was offered through the parent study protocol, mainly to adolescent girls and young women (AGYW), and the parent study team amended protocols to include data and sample collection for HIVDR in individuals who seroconverted on PrEP (Supplement 1).

All participants provided written informed consent at the time of HIV‐1 diagnosis. Details of the adaptable protocol template, sample transport logistics and supportive materials used for implementation have been published previously [35].

### 2.2 Study procedures

Clinics and sites offering PrEP alerted the GEMS study coordinator when an individual on PrEP was diagnosed with HIV‐1 according to their national HIV‐1 testing algorithm. Individuals were eligible for participation if: (1) they were a current PrEP user, defined as an individual who has collected an initial supply of PrEP agents or a resupply of PrEP agents at any time in the past (Kenya) or in the last 3 months (Eswatini, Zimbabwe and specific projects in South Africa), independent of self‐reported adherence; (2) identified as having acquired HIV‐1, as per the HIV‐1 Testing Algorithm in the National Guidelines of each country; and (3) aged 15 years or older in Kenya, aged 16 years or older in Eswatini or of age to receive PrEP in Zimbabwe. To enrol participants, healthcare workers used a GEMS‐provided kit that contained consent documents, case report forms and material needed for blood collection. After consent, a brief clinical history was collected to assess each participant's risk of HIV‐1 acquisition and self‐reported PrEP adherence.

Sample collection varied by site. Dried blood spots (DBS) prepared at the local clinic or unprocessed whole blood was transported to a central laboratory within 48–72 hours in each country for processing, storage and/or shipment. In Kenya, samples were sent to the WHO HIVDR testing laboratory at the Kenya Medical Research Institute (KEMRI) in Kisumu; samples from South Africa and Eswatini were sent to BARC‐Lancet Laboratories in Johannesburg; and samples from Zimbabwe and DBS and/or plasma that could not be tested locally were sent to the University of Pittsburgh–U.S. Whole blood was processed to plasma and DBS for HIV‐1 RNA, HIVDR, and drug‐level testing at the destination laboratory. Service providers received standardized training for all study procedures.

### 2.3 Laboratory methods

#### 2.3.1 HIV‐1 RNA

Levels of HIV‐1 RNA from DBS were quantified using a modified assay where one or two spots were extracted into either a manufacturer‐supplied buffer or 1X phosphate‐buffered saline, and then run on a commercial assay (Hologic Aptima HIV‐1 Quant Dx, DBS limit of detection [LOD] 883 copies/ml; Abbott RealTime HIV‐1 Viral Load, DBS LOD 839 copies/ml; or Roche COBAS TaqMan HIV‐1 TEST v2.0, DBS LOD 400 copies/ml) according to the manufacturer's instructions. HIV‐1 RNA from plasma was quantified using Abbott RealTime (LOD 40 copies/ml) or Roche TaqMan (LOD 20 copies/ml).

#### 2.3.2 Tenofovir‐diphosphate levels

Tenofovir‐diphosphate (TFV‐DP) was quantified as an objective adherence measure from DBS at the University of Cape Town or the University of Colorado, as described previously [36]. Briefly, a 25 µl punch was taken from a DBS card and extracted with methanol:water and isotopic tenofovir as an internal standard. Colorado used an updated methanol:water extraction procedure that extracted slightly more drug from the DBS card, resulting in higher concentrations [37, 38]. TFV‐DP was quantified by validated liquid chromatography/tandem mass spectrometry (LC/MS). The original assay (performed by University of Cape Town) was used for interpretation benchmarks as follows: TFV‐DP levels <350 femtomoles per punch (fmol/punch) or below limit of quantitation (BLQ) were categorized as “Low”; TFV‐DP levels between 350 and 699 fmol/punch were categorized as “Moderate”; and TFV‐DP levels ≥700 fmol/punch were categorized as “High.”

#### 2.3.3 HIV‐1 drug resistance testing

HIV‐1 genotyping was performed by population‐based Sanger sequencing, with the exact methodology differing by lab. HIV‐1 RNA results were not always available prior to testing for drug resistance; all samples were attempted and were not censored based on a predefined RNA threshold. At KEMRI in Kenya, HIV‐1 RNA was extracted from plasma, or total nucleic acid was extracted from DBS by NucliSENS® easyMag® (bioMérieux) and then amplified and sequenced using the Applied Biosystems HIV‐1 Genotyping Kit (*protease* and *reverse transcriptase* only) (ThermoFisher Scientific). At BARC‐Lancet in South Africa, a validated in‐house assay was used with plasma HIV‐1 RNA extraction by guanidinium thiocyanate lysis/isopropanol precipitation and primers optimized for non‐B HIV‐1 subtypes, as previously described [39].

For DBS sent to the University of Pittsburgh, a modified laboratory‐developed short‐amplicon assay was performed that targeted *reverse transcriptase* codons 58–197 for specific coverage of mutations known to be associated with TDF and/or XTC resistance. From each donor, total nucleic acid was extracted from one DBS using the automated bioMérieux NucliSENS® easyMag® extraction system according to WHO guidance and was eluted in 30 µl elution buffer [40]. Reverse transcription–polymerase chain reaction (RT‐PCR) amplification was performed with 18 µl of extracted nucleic acid and using the SuperScript™ III One‐Step RT‐PCR System (ThermoFisher) and touchdown PCR during the PCR phase of the reaction [41, 42]. An additional PCR amplification with semi‐nested primers was performed using 1 µl of the RT‐PCR product, followed by exoSAP (ThermoFisher) purification and Sanger sequencing (Azenta Life Sciences).

FASTA files were concatenated from all resistance tests and analysed together to identify resistance‐associated mutations and viral subtypes using the Stanford Genotypic Resistance Interpretation Algorithm v9.6 [43, 44, 45]. PrEP‐associated mutations are defined as amino acid changes at the following codons in HIV‐1 *reverse transcriptase*: K65R, K70E, M184I and/or M184V in HIV‐1 reverse transcriptase.

#### 2.3.4 Statistical analysis

Descriptive analyses of the study sample characteristics overall and by country included frequencies and percentages for categorical demographic, HIV risk, and HIVDR mutation variables and means and 95% confidence intervals for continuous variables, including time on PrEP prior to HIV‐1 diagnosis. The frequency of self‐reported adherence was summarized by TDF levels, HIV‐1 RNA levels and HIV‐1 seroconversions with drug resistance‐associated mutations.

## 3 RESULTS

**Figure 1:**
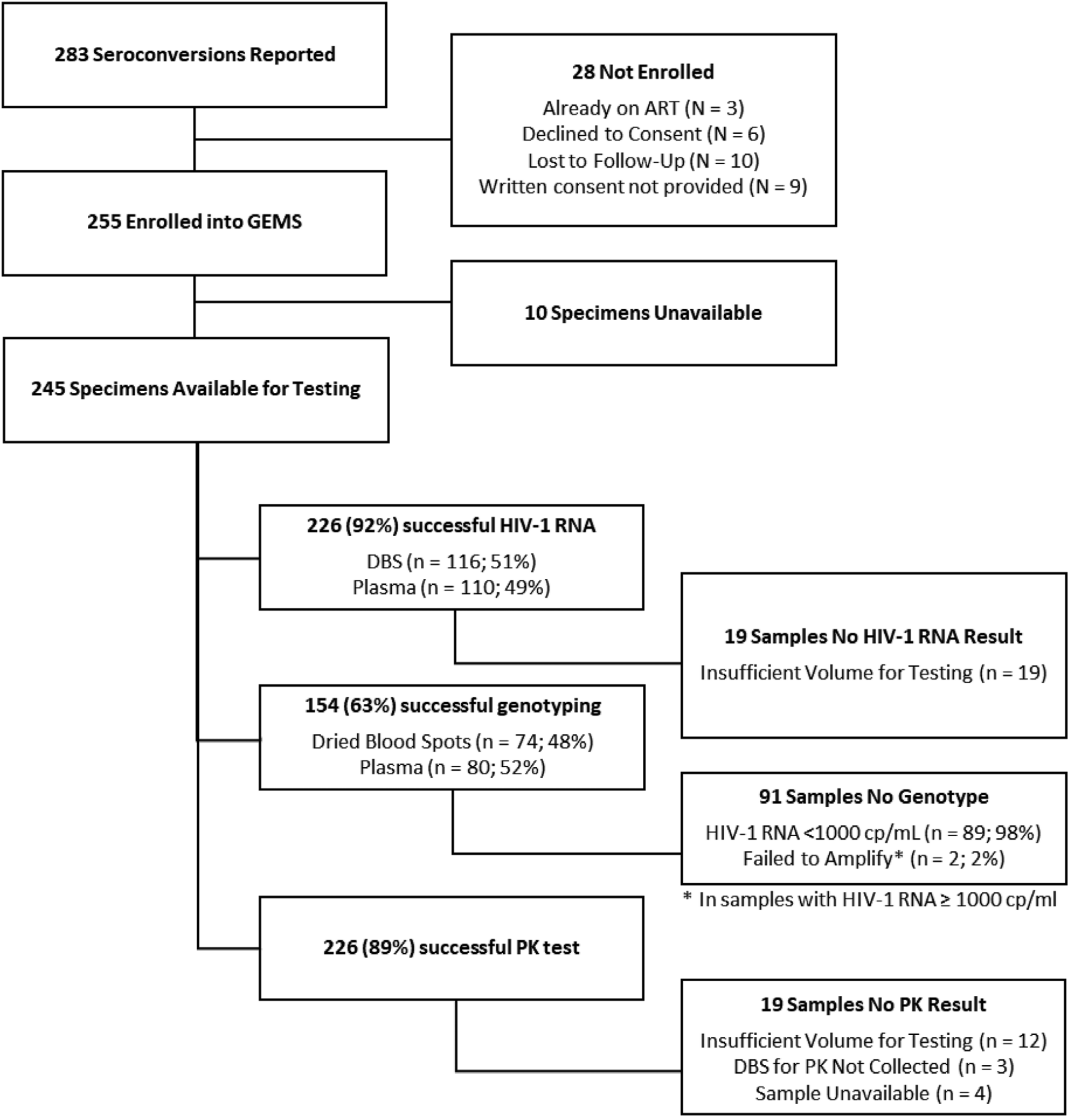
Consort diagram. Abbreviations: ART, antiretroviral therapy; cp/ml, copies per millilitre; DBS, dried blood spots; GEMS, Global Evaluation of Microbicide Sensitivity; HIV‐1, human immunodeficiency virus type 1; *N*, number; PK, pharmacokinetic; RNA, ribonucleic acid.

From July 2017 through September 2023, 283 seroconversions on PrEP were reported from three countries conducting national protocols (Kenya, Zimbabwe and Eswatini) and one country through implementing partner projects (South Africa). Of these, 255 (90%) individuals consented to participate. Reasons for not enrolling included: three individuals who had already initiated ART and were outside the enrolment window, six who declined to consent, 10 who could not be reached after confirmed seroconversion and nine who provided oral but not written consent, therefore, were not enrolled into the study. Specimens from 245 participants were available for determining HIV‐1 RNA levels, standard genotype for HIV‐1 drug resistance detection and pharmacokinetics for estimating adherence to the PrEP product; 10 collected specimens were lost before testing could be completed (Figure 1).

### 3.1 Demographics and study‐related characteristics

**Figure 2:**
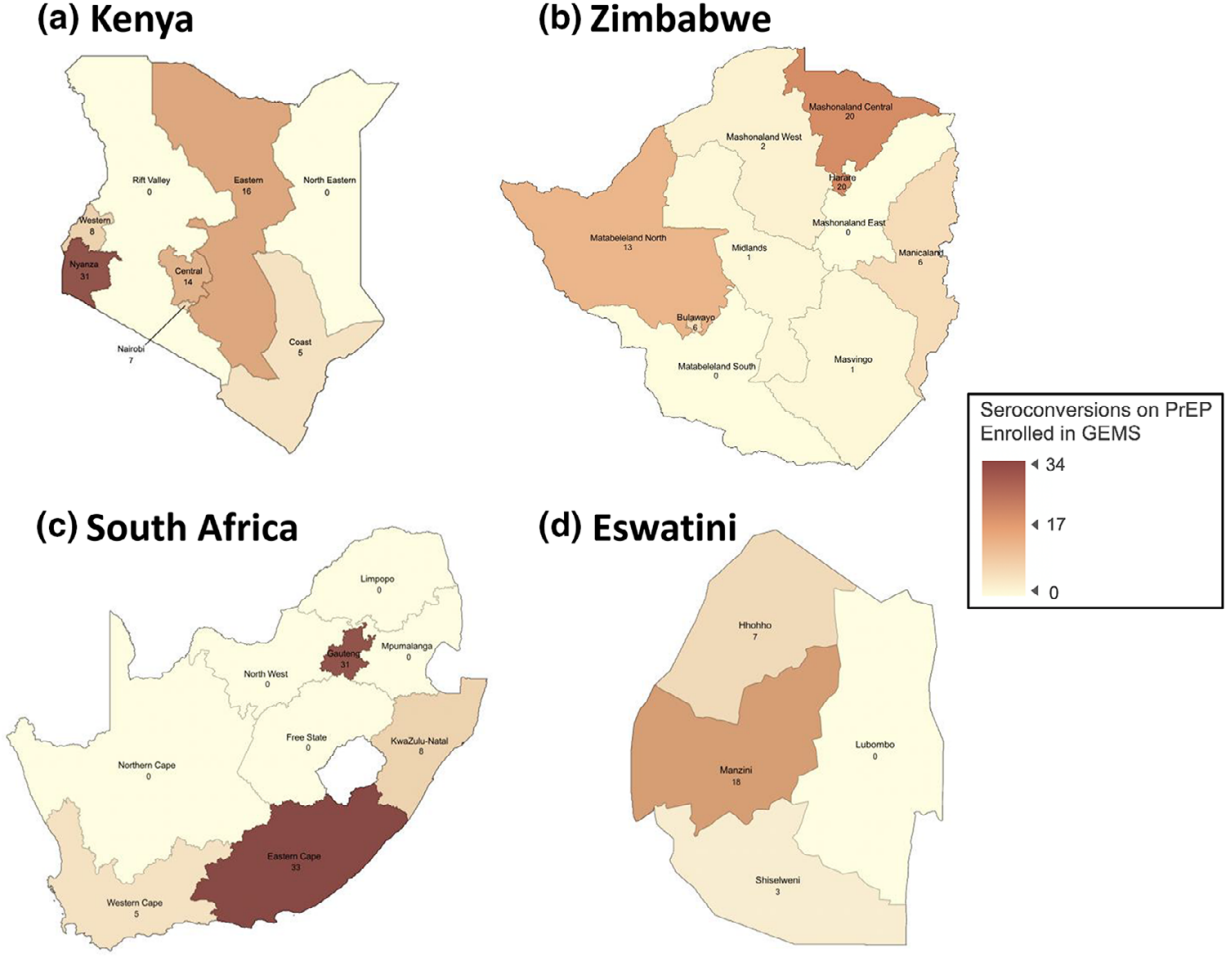
Geographic distribution of reported seroconversions on tenofovir disoproxil fumarate/emtricitabine or lamivudine oral pre‐exposure prophylaxis enrolled in the Global Evaluation of Microbicide Sensitivity (GEMS) Project (2017–2023). *Note*: Shading is proportionate to the number of cases of reported seroconversion on oral pre‐exposure prophylaxis, with darker shading indicating more cases. Maps were created using ArcGIS [58, 59].

Participants were predominantly female (193 of 255; 76%), and 12 (5%) were transgender women. Half of the participants were younger than 25 (130 of 255; 51%). Participants self‐reported their reasons for taking PrEP as being in a serodifferent partnership (25%), being a female sex worker (8%) or a man having sex with men (5%), having sex with unknown partners (5%), being a pregnant or lactating individual (4%) or incarceration (1%). The 255 participants were enrolled from Kenya (81; 32%), South Africa (77; 30%), Zimbabwe (69; 27%) and Eswatini (28; 11%) (Table 1). Participants were referred from both urban and rural clinics with disparate geographic distribution (Figure 2).

**Table 1:** Characteristics of study participants

Variable	Kenya *N* = 81	South Africa *N* = 77	Zimbabwe *N* = 69	Eswatini *N* = 28	Total *N* = 255
Date range of reporting	03/2018–01/2023	12/2017–01/2021	09/2018–09/2023	01/2020–06/2021	12/2017–09/2023
Sex					
Female	59 (73%)	58 (75%)	50 (72%)	24 (86%)	193 (76%)
Male	21 (26%)	8 (10%)	17 (25%)	4 (14%)	50 (20%)
Transgender (MTF)	0 (0%)	11 (14%)	1 (1%)	0 (0%)	12 (5%)
Unknown	1 (1%)	0 (0%)	1 (1%)	0 (0%)	2 (1%)
Age at seroconversion					
15–24 years	26 (32%)	56 (72%)	32 (46%)	14 (50%)	130 (51%)
25–34 years	30 (37%)	19 (25%)	22 (32%)	11(39%)	82 (32%)
>35 years	20 (26%)	2 (3%)	14 (20%)	3 (11%)	40 (16%)
Unknown	4 (5%)	0 (0%)	1 (1%)	0 (0%)	5 (2%)
HIV‐1 self‐reported reason for PrEP					
Serodifferent couple	44 (54%)	0 (0%)	14 (20%)	6 (21%)	64 (25%)
Female sex worker	7 (9%)	5 (6%)	9 (13%)	0 (0%)	21 (8%)
MSM	5 (6%)	0 (0%)	9 (13%)	0 (0%)	14 (5%)
High risk^a^	10 (12%)	0 (0%)	2 (3%)	1 (4%)	13 (5%)
Pregnant and/or lactating	1 (1%)	0 (0%)	0 (0%)	8 (29%)	9 (4%)
Incarcerated	0 (0%)	0 (0%)	1 (1%)	1 (4%)	2 (1%)
Unknown	8 (10%)	19 (25%)	14 (20%)	7 (25%)	48 (19%)

Abbreviations: HIV‐1, human immunodeficiency virus type 1; MSM, men who have sex with men; MTF, male to female; *N*, number; PrEP, pre‐exposure prophylaxis.

^a^ Self‐defined as high risk; includes unknown partner.

### 3.2 Time on PrEP before HIV‐1 diagnosis

We evaluated PrEP initiation and HIV‐1 diagnosis dates to assess the duration of time individuals were on PrEP before acquiring HIV‐1. Of 255 participants, dates were missing for 10 individuals because the date of PrEP initiation and/or date of HIV‐1 diagnosis was not recorded. Of the remaining 245 participants, 83 (34%) seroconverted within 90 days of initiating PrEP; 34 of those 83 seroconverted within 30 days of initiating PrEP, which suggests that up to one‐third of individuals initiating PrEP could have been in the acute phase of HIV‐1 infection prior to starting PrEP or could have acquired HIV‐1 soon after PrEP initiation (Table 2). We also evaluated the duration of time between reported seroconversion and sample collection. The specimen collection and/or HIV‐1 diagnosis date was not documented for five of 245 samples available for testing. Most specimens (180; 73%) were collected within 2 weeks of HIV‐1 diagnosis, and 50 (20%) were collected within 2 weeks to 3 months of diagnosis.

**Table 2:** Time on PrEP before HIV‐1 seroconversion

Days between PrEP initiation and reported HIV‐1 seroconversion	Number of Individuals (%) *N* = 245^a^	Mean (95% CI) in days
0–90 days (0–3 months)	83 (34%)	44 (40, 48)
91–180 days (3–6 months)	56 (23%)	135 (128, 142)
181–365 days (6–12 months)	56 (23%)	242 (230, 256)
>365 days (>1 year)	50 (20%)	628 (554, 701)

^a^ Date of pre‐exposure prophylaxis (PrEP) initiation and/or date of reported HIV‐1 seroconversion was not available for 10 individuals.

### 3.3 PrEP adherence by self‐report and intracellular drug levels

**Figure 3:**
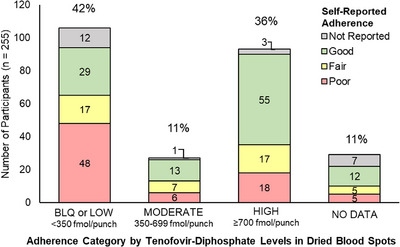
Number (%) of participants with detectable tenofovir‐diphosphate levels in dried blood spots, categorized by self‐reported adherence. *Note*: The percent above each bar is calculated as the proportion in each category with respect to total participants (*n* = 255). Tenofovir‐diphosphate (TFV‐DP) levels were measured in dried blood spots using mass‐spectrometry/liquid chromatography and were categorized as “Low” if TFV‐DP levels were <350 femtomoles per punch (fmol/punch) or below limit of quantitation (BLQ); “Moderate” if TFV‐DP was 350–699 fmol/punch; and “High” if TFV‐DP was ≥700 fmol/punch. Self‐reported adherence was collected on case report forms during the clinical intake at sites in Kenya, Eswatini, Zimbabwe and South Africa. “Good” was defined as “Missed 0–3 doses in the past month” (Kenya only) or “Usually used medicine as instructed”; “Fair” was defined as “Missed 4–5 doses in the last month” (Kenya) or “Used medication but not daily”; “Poor” was defined as “Missed 6–7 doses or more in the last month” (Kenya), “Mostly did not use medicine as instructed” and/or “Stopped taking PrEP completely” (Eswatini only).

We estimated PrEP adherence from self‐reports in 232 of 255 (91%) participants and from TFV‐DP drug concentration measurements from DBS collected at the time of study enrolment in 226 of 255 participants (89%). Of the 226 participants with drug‐level data, 93 (41%) acquired HIV‐1 despite having high concentrations of TFV‐DP (mean 1579 ± 717; range 717–5922 femtomoles per punch [fmol/punch]); 55 of the 93 (59%) also reported having “good” adherence, defined as missing 0–3 doses in the last month, or usually taking medicine as instructed. Thirty‐eight of 226 participants (17%) had low TFV‐DP concentrations (mean 177 ± 91; range 27–343 TFV‐DP fmol/punch), and 68 of 226 (30%) had unquantifiable concentrations of TFV‐DP (below LOD quantification; <31.25 or <16.6 fmol/punch). Of the 106 participants with low or unquantifiable TFV‐DP, 65 (61%) self‐reported “poor” or “fair” adherence (Figure 3).

### 3.4 HIV‐1 RNA levels at seroconversion

Of the 245 specimens available for testing, 128 (52%) were DBS and 117 (48%) were plasma. HIV‐1 RNA levels were not available for 19 specimens (12 DBS and 7 plasma) due to insufficient sample. Of the 226 remaining specimens, 114 (50%) had undetectable HIV‐1 RNA; 75 of 116 (65%) DBS (LOD 400, 839 or 883 copies/ml), and 39 of 110 (35%) plasma (LOD 20, 40 or 400 copies/ml, depending on the local laboratory assay used). Of 112 samples with detectable HIV‐1 RNA, 29 (26%) samples had <200 copies/ml of HIV‐1 RNA (median 102; range 23–196) and 83 (74%) had ≥200 copies/ml HIV‐1 RNA (median 4000; range 209–127,000) (Figure 1 and Table 4).

### 3.5 Seroconversions on oral tenofovir disoproxil fumarate/emtricitabine or lamivudine PrEP with drug resistance‐associated mutations

Of the 245 samples available for testing, 154 (63%) were successfully genotyped. The primary reason for no result was low levels of HIV‐1 RNA (89 of 91 samples with HIV‐1 RNA <1000 copies/ml) (Figure 1). Overall, 34 of 154 (22%, 95% CI [16%, 29%]) seroconversions on oral PrEP that were successfully genotyped had PrEP‐associated NRTI mutations (amino acid changes at codons 65, 70 and/or 184); 16 of 34 (47%) had PrEP‐associated mutations only; and 18 of 34 (53%) had PrEP‐associated mutations with non‐nucleoside reverse transcriptase inhibitor (NNRTI) mutations (Table 3). Of the 32 individuals with PrEP‐associated resistance and drug concentration results, the majority (24 of 32, 75%) had TFV‐DP levels consistent with high adherence, whereas 7 of 32 (22%) had TFV‐DP levels consistent with low or moderate adherence. TDF is a common component of first‐line ART, therefore, detection of TFV‐DP could reflect PrEP or ART use. Samples were predominantly subtype C (26 of 34, 76%); six individuals had subtype A or A1 HIV‐1, and two individuals had subtype D HIV‐1. NNRTI mutations observed included L100I, K101E, K103SN, V106IM, E138AQ, V179T, Y188L and G190A (Table 4).

**Table 3:** Individuals with HIV‐1 drug resistance mutations by drug class as a proportion of total number of seroconversions with drug resistance results per country

Mutation profile	Kenya *N* = 47	South Africa *N* = 54	Zimbabwe *N* = 37	Eswatini *N* = 16	Total *N* = 154
No major mutations in HIV‐1 RT^a^	26 (55%)	29 (54%)	18 (49%)	10 (63%)	83 (54%)
PrEP‐associated RT mutations only^b^	3 (6%)	9 (17%)	2 (5%)	2 (13%)	16 (10%)
PrEP‐associated RT mutations with NNRTI mutations^b^	5 (11%)	5 (9%)	8 (22%)	0 (0%)	18 (12%)
NNRTI mutations only^c^	13 (28%)	11 (20%)	9 (24%)	4 (25%)	37 (24%)

Abbreviations: HIV‐1, human immunodeficiency virus type 1; *N*, number; NNRTI, non‐nucleoside reverse transcriptase inhibitor; PrEP, pre‐exposure prophylaxis; RT, *reverse transcriptase*.

^a^ Major mutations were defined by the Stanford University Drug Resistance Database, v9.6 [43, 44, 45].

^b^ PrEP‐associated mutations are defined as amino acid changes at codons 65, 70 and/or 184 in HIV‐1 *reverse transcriptase*.

^c^ Three individuals also had single thymidine analogue mutations each, unrelated to PrEP, as follows: (1) K219Q, (2) T215F and (3) E44D.

**Table 4:** HIV‐1 reverse transcriptase genotype and tenofovir‐diphosphate levels of individuals who seroconverted on oral disoproxil fumarate/emtricitabine pre‐exposure prophylaxis (PrEP) with PrEP‐associated mutations

PID	HIV‐1 RNA cp/ml	Sub‐type	NRTI	NNRTI	TDF‐DP fmol/punch (interpretation)^a^
**Kenya**					
KE1*	4224	A1	M184V	−	1096 (HIGH)
KE2	ND^b^	A1	M184MIV	−	ND^b^
KE3*	ND^b^	A1	M184MIV	−	ND^b^
KE4*	23,100	A1	M184V	K103N	792 (HIGH)
KE5	ND^b^	A1	L74I, M184V, K219Q	K103N, V179T, G190A	1567 (HIGH)
KE6*	<883	A1	K65KR, K70KE, M184V	V106IV, V179T	ND^b^
KE7*	ND^b^	D	K70N, L74I, M184V	K103S, G190A	1270 (HIGH)
KE8*	ND^b^	D	M184V	E138A, G190A	1145 (HIGH)
**South Africa**					
SA1	134	C	M184V	−	739 (HIGH)
SA2	<40	C	M184V	−	1680 (HIGH)
SA3	916	C	M184V	−	221 (LOW)
SA4	TND^c^	C	K65R, K70KE, M184V	−	1116 (HIGH)
SA5*	11,458	C	M184MIV	−	1247 (HIGH)
SA6	2349	C	M184MV	−	265 (LOW)
SA7	160	C	M184V	−	956 (HIGH)
SA8	361	C	M184MIV	−	442 (MOD)
SA9	<40	C	M184MIV	−	132 (LOW)
SA10	10,827	C	M184V	K103N	998 (HIGH)
SA11	<40	C	M184MV	E138A	634 (MOD)
SA12	TND^c^	C	M184V	E138A	787 (HIGH)
SA13	762	C	K70E, M184V	V106M	876 (HIGH)
SA14	43	C	M184V	V106M	1737 (HIGH)
**Zimbabwe**					
ZB1*	<400	C	M184V	−	1097 (HIGH)
ZB2*	700	C	M184V	−	1077 (HIGH)
ZB3*	<883	C	M184V	V106M	1242 (HIGH)
ZB4*	15,124	C	M184MI	K103N	1108 (HIGH)
ZB5*	95,700	C	K65R, M184V	K101E, V106M, E138A, G190A	4219 (HIGH)
ZB6*	<883	C	M184V	K103N	645 (MOD)
ZB7*	760	C	M184V	E138A	691 (MOD)
ZB8*	139,814	C	K65R, M184V	E138A	1181 (HIGH)
ZB9*	22,200	C	K65R, M184V	L100I, K103N	1160 (HIGH)
ZB10*	<400	C	K65R, M184V	V106M, E138Q, Y188L	1287 (HIGH)
**Eswatini**					
EW1^d^, ^**^	<20	C	K65KR	K103N	1172^e^ (HIGH)
EW2	440	C	M184I	−	2086^e^ (HIGH)

^a^ TFV‐DP pharmacokinetic drug levels are interpreted as follows: <350 fmol/punch as low adherence (LOW); 350–699 fmol/punch as moderate adherence (MOD); ≥700 fmol/punch as high adherence (HIGH). No individuals had drug levels that were below the limit of quantitation.

^b^ Not done (ND) due to insufficient sample volume or sample unavailable.

^c^ Target not detected (TND) with limit of detection 40 HIV‐1 RNA copies/ml.

^d^ Participant was reported to be on antiretroviral therapy for 107 days prior to sample collection.

^e^ Pharmacokinetic drug levels were also performed at a second lab, with results of 2550 fmol/punch (EW1) and 2814 fmol/punch (EW2).

* HIV‐1 RNA and genotype done using DBS.

** HIV genotype done using DBS.

Abbreviations: cp/ml, copies per millilitre; fmol/punch, femtomole per dried blood spot punch; HIV‐1, human immunodeficiency virus type 1; NNRTI, non‐nucleoside reverse transcriptase inhibitor; NRTI, nucleoside or nucleotide reverse transcriptase inhibitor; PID, participant identifier; RNA, ribonucleic acid; TDF‐DP, tenofovir diphosphate.

## 4 DISCUSSION

More than 100,000 individuals initiated PrEP in national rollout and/or PrEP delivery projects in Kenya, South Africa, Zimbabwe and Eswatini from 2017 through 2023. GEMS is among the largest studies of reported seroconversions on PrEP (*n* = 283) outside of clinical trials, and one of the first to describe HIVDR frequency in a unique population of individuals who accessed PrEP programmatically across three countries or in targeted population‐based rollout in South Africa. GEMS was highly successful in demonstrating the feasibility of using routine care sites in diverse geographical settings without research experience to administer a brief consent and conduct study procedures and the willingness of individuals (90% of all reported seroconversions) to enrol in a study and provide a blood sample immediately after learning of their HIV‐1 seroconversion despite being on PrEP.

GEMS worked closely with ministries of health and local partners to incorporate HIVDR monitoring into technical working group discussions and national PrEP plans [46, 47]. Extensive effort was made to raise awareness of the national protocol through country‐level trainings embedded in larger PrEP implementation training‐of‐trainer sessions. GEMS kits containing all the materials needed to support enrolment and sample collection were widely distributed at trainings through county Acquired Immunodeficiency Syndrome (AIDS) and Sexually Transmitted Infections (STI) coordinators in Kenya and through implementing partners [48]. The representativeness of the 283 seroconversions in GEMS is not known because the actual number of seroconversions on PrEP that occurred in partner countries is unknown, and underreporting of seroconversions is likely.

Half of participants had undetectable HIV‐1, which may be due to a much higher LOD for DBS (550–1000 copies/ml as compared to 20–50 copies/ml for plasma) [49], RNA degradation during ambient temperature transport, reduction in viral replication due to continued use of PrEP or participants already having initiated ART. In our dataset, ART initiation dates were not available for most participants, although most are expected to have started ART on the day of diagnosis per national policy. Only seven participants with known ART initiation dates had HIVDR test results, and only one of those seven had PrEP‐related mutations, as noted in Table 4. Thus, the results are expected to reflect pre‐treatment drug resistance rates after seroconversion on PrEP, with the limitation that these were community‐acquired samples and not samples from a controlled study.

The rate of PrEP‐associated HIVDR observed (22%) is consistent with the overall rate reported by other implementation studies and is higher than those observed in clinical trials of PrEP [13, 14, 15]. Of the 245 individuals reported to this study to have acquired HIV on PrEP, 33% had seroconverted within 3 months of initiating PrEP, suggesting that some individuals may have started PrEP during the acute phase of HIV‐1 acquisition when resistance risk is highest. Unlike in trials, stored retrospective samples to determine HIV status at PrEP initiation were unavailable. The rate of NNRTI resistance in this population was also high; 24% of individuals who acquired HIV‐1 had NNRTI resistance alone, indicating that the resistance was transmitted from a partner, and an additional 12% had PrEP‐associated NRTI resistance with NNRTI resistance, which could have been transmitted from a partner, partially selected by PrEP or both. All individuals with both PrEP‐associated resistance and drug concentration results had quantifiable TFV‐DP at varying concentrations, indicating drug levels may have been inadequate to prevent HIV‐1 acquisition but sufficient to exert selection pressure for HIV‐1 to develop resistance, as has been observed for other PrEP agents [50, 51].

First‐line tenofovir‐lamivudine‐dolutegravir (TLD) will likely be effective in achieving virologic suppression in individuals with M184I/V and/or K65R, based on findings from the NADIA study that demonstrated high rates of viral suppression with TLD even in individuals with M184V/I or K65R [52]. In studies of the dual‐combination therapy lamivudine (3TC) plus dolutegravir (DTG), the probability of viral rebound was not statistically significant in individuals who had HIV‐1 with or without M184V. However, the duration of viral suppression was found to be shorter in individuals who had HIV‐1 with M184V [53]. Teyssou and colleagues characterized M184V decrease kinetics in proviral DNA in people living with HIV‐1 and found that archived M184V was undetectable in 50% of individuals at 2.5 years after the first observed time point, independent of the type of ART taken [54]. Regulatory approval and scale‐up of TLD was just beginning in many countries between 2016 and 2019, therefore, some individuals may have started on older NNRTI‐based first‐line regimens if TLD was unavailable at the time of their diagnosis with HIV‐1. Long‐term outcomes in individuals on TLD with reduced susceptibility to tenofovir and/or lamivudine also remain unknown. Low but rising rates of dolutegravir resistance in individuals on failing TLD have been reported, and the accumulation of integrase strand transfer resistance can lead to higher levels of resistance to dolutegravir [55, 56].

A meta‐analysis of 18 studies demonstrated a significant protective effect with higher PrEP adherence [57]. Although 41% of participants had high TFV‐DP levels detected and/or self‐reported good adherence, their drug levels may be elevated and not reflective of adherence at the time of HIV‐1 acquisition if they had taken PrEP in the time leading up to providing a sample. With a same‐day ART start, the possibility that ART had been taken prior to providing a sample also cannot be ruled out. Tenofovir is a common component in both PrEP and first‐line ART regimens. Adherence measures were cross‐sectional and available only from a DBS at the time of sample collection at HIV diagnosis; thus, adherence may have been underestimated if TFV‐DP levels had not reached steady state. Finally, the relationship between self‐report and TFV‐DP for adherence may have been influenced by different adherence scales for the measures. Pre‐seroconversion longitudinal pharmacokinetics (PK) sampling at multiple time points would be a more accurate indicator of PrEP adherence, which was not available for this study.

This study had several additional limitations. Resistance monitoring was done through passive surveillance at clinics delivering PrEP, and not through comprehensive surveillance; thus, the true number of PrEP users and PrEP seroconversions in partner countries is uncertain. Clients were asked to self‐identify as a PrEP user, defined as having collected an initial supply of PrEP agents or a resupply of PrEP agents at any time in the past (Kenya) or in the last 3 months, independent of self‐reported adherence, in Eswatini, Zimbabwe and some projects in South Africa. Objective verification of prior PrEP use (such as by prescription records) was not available. Despite these limitations, GEMS provided a comprehensive assessment of resistance risk with seroconversion during the rollout of oral PrEP in programmatic settings.

## 5 CONCLUSIONS

Oral PrEP remains an important and highly effective component of combination HIV prevention, and the number of individuals protected by PrEP far exceeds the relatively low number of people acquiring HIV‐1 on PrEP that have been reported. Overall, 32 (22%) of 154 seroconversions on oral PrEP with successful genotypes had PrEP‐associated resistance mutations (K65R and/or M184I/V) with or without NNRTI mutations; these individuals are likely to achieve virologic suppression with TLD, although long‐term outcomes remain undefined. HIVDR is a risk when PrEP is: (1) started during the acute phase of HIV‐1 acquisition; (2) transmitted by a partner with resistant HIV‐1; (3) used with suboptimal adherence and drug levels that allow HIV‐1 to breakthrough with selection of resistance. In the context of rising pre‐treatment resistance, improved identification of acute HIV‐1 infection before PrEP initiation and monitoring for PrEP‐related resistance are important for optimizing antiretroviral use for both treatment and prevention. These data highlight the importance of ongoing surveillance to assess rates of pre‐treatment drug resistance to ensure preservation of drugs used for both HIV‐1 treatment and prevention, particularly in the context of new PrEP methods, all of which are antiretroviral‐based.

## COMPETING INTERESTS

UMP serves as a consultant to Merck, separate from this work. PLA receives grant funding from Gilead Sciences, Inc. to the University of Colorado (unrelated to the current work). CC has served as a scientific advisor to Gilead, Merck and GSK, separate from this work. BAR serves as a DSMB member for Gilead Sciences clinical trials, separate from this work. JWM is a consultant to Gilead Sciences, Inc. and has received grant funding from Gilead Sciences, Inc. to the University of Pittsburgh (unrelated to the current work); receives compensation from Galapagos NV (unrelated to the current work); and holds share options in Galapagos NV, Infectious Disease Connect, Inc. and MingMeg Biotechnology Co., Ltd. (unrelated to the current work).

## AUTHORS’ CONTRIBUTIONS

UMP, LDK and JWM wrote the original draft, conceptualized the project and provided leadership to the project. LL, JMP and KT provided all policy‐based leadership, including writing and gaining approvals for protocols and monitoring and evaluations. The following individuals provided project management, investigation and execution: in Kenya (EB, BC, IM, SM), Zimbabwe (NN, IM, OM, GN), Eswatini (AH, SNM), South Africa (SM, CLW) and multiple countries (CC). ALH, KJP, KDM, CLW, LW and PLA conducted laboratory‐based methods development and research design for sample testing. BAR provided statistical analysis. DC and SA provided oversight and guidance for the project. All authors reviewed and edited the final manuscript.

## FUNDING

This project was made possible by the generous support of the American people through the United States Agency for International Development (USAID), in partnership with the President's Emergency Plan for AIDS Relief (PEPFAR), under the terms of Cooperative Agreement numbers AID‐OAA‐A‐15‐00031 and 7200AA21CA00011 until 23 January 2025.

## DISCLAIMER

The contents of this paper are the responsibility of the University of Pittsburgh and do not necessarily reflect the views of USAID or the United States Government.

## AUTHOR INFORMATION

The following authors have current affiliations that are different than those at the time of the study: Everline Bosek, Irene Mukui, Sarah Masyuko, Nonhlanhla Ndlovu, Anita Hettema, Carole L. Wallis, Kevin D. McCormick, Jill M. Peterson, Shannon Allen, and Kristine Torjesen.

## Supporting information




**Supplement** **1** **: Global Evaluation of Microbicide Sensitivity Partner Projects**. Details of Institutional Review Board and/or Ethics Committee approvals for projects that partnered with GEMS and provided data and/or samples are provided in this supplement.

## Data Availability

The data that support the findings of this study are available on request from the corresponding author. The data are not publicly available due to privacy or ethical restrictions. De‐identified raw HIV‐1 *pol* sequences will be deposited in the National Center for Biotechnology Information GenBank (https://www.ncbi.nlm.nih.gov/genbank/) upon publication.
