# Supplementary material for: A cross‐sectional study evaluating the frequency of HIV drug resistance mutations among individuals diagnosed with HIV‐1 in tenofovir disoproxil fumarate‐based pre‐exposure prophylaxis rollout programmes in Kenya, Zimbabwe, Eswatini and South Africa
Source: J Int AIDS Soc. 2025 Aug 20;28(8):e70011. doi: 10.1002/jia2.70011 (PMC12367864; doi:10.1002/jia2.70011)
Supplement: Supplementary file 1 — Supplement 1 : Global Evaluation of Microbicide Sensitivity Partner Projects. Details of Institutional Review Board and/or Ethics Committee approvals for projects that partnered with GEMS and provided data and/or samples are provided in this supplement. [file JIA2-28-e70011-s001.docx]

**Supplement 1**. **Global Evaluation of Microbicide Sensitivity (GEMS) Ethics Approvals and Partner Projects**

1. **National Protocols**

**A1. Kenya**

| Country | Kenya |
| --- | --- |
| Implementing Partners | National AIDS and STI Control Program (NASCOP)  University of Washington-Kenya |
| Title of Protocol | A Comprehensive Assessment of HIV Drug Resistance among Seroconverters using Tenofovir Disoproxil Fumarate-Containing Pre-Exposure Prophylaxis in Kenya |
| Approval Number | Kenya Medical Research Institute Scientific and Ethics Review Unit through the Center for Viral Research KEMRI/SERU/CVR/018/ 3636 |
| Approval Date | 5 March 2018 |
| Age Eligibility | 15 years and older |
| HIV-1 Testing Schedule | Current national HIV testing algorithm |

**A2. Zimbabwe**

| Country | Zimbabwe |
| --- | --- |
| Implementing Partners | AIDS and TB Programme Zimbabwe Ministry of Health and Child Care  Pangaea Zimbabwe |
| Title of Protocol | An Assessment of HIV Drug Resistance among Seroconversions in Users of Pre-Exposure Prophylaxis in Zimbabwe |
| Approval Number | Medical Research Council of Zimbabwe MRCZ/A/2309 |
| Approval Date | 6 September 2018 |
| Age Eligibility | Of age to receive PrEP |
| HIV-1 Testing Schedule | Current national HIV testing algorithm |

**A3. Eswatini**

| Country | Eswatini |
| --- | --- |
| Implementing Partners | Eswatini National AIDS Program  Clinton Health Access Initiative (CHAI) |
| Title of Protocol | An Assessment of HIV Drug Resistance among Seroconversions in Users of Pre-Exposure Prophylaxis in Eswatini |
| Approval Number | Eswatini Health and Human Research Review Board Registration Number FWA 00026661/IRV 00011253; Protocol Number SHR199/2019 |
| Approval Date | 23 January 2020 |
| Age Eligibility | 16 years and older |
| HIV-1 Testing Schedule | Current national HIV testing algorithm |

1. **Partner Projects**

**B1. Project PrEP**

| Country | South Africa |
| --- | --- |
| Implementing Partner | Wits RHI |
| Title of Protocol | The Introduction of Pre-Exposure Prophylaxis (PrEP) into Comprehensive Sexual and Reproductive Health Services for Adolescent Girls and Young Women (AGYW) in South Africa: An Implementation Science Study |
| Approval Number | World Health Organization (WHO) Ethics Review Committee (ERC) Review: Wits PrEP-AGYW-Main Protocol 0003088; Wits RHI Human research ethics committee (HREC) Reference Number M180860 |
| Approval Date | 6 August 2018 |
| Age Eligibility | 15 years and older |
| HIV-1 Testing Schedule | Two rapid antibody tests at PrEP initiation, 1-month visit and every 3 months^a^ |

**B2. WITS-RHI Key Populations Programme**

| Country | South Africa |
| --- | --- |
| Implementing Partner | Wits RHI |
| Title of Protocol | HIV Drug Resistance Assessment in PrEP Seroconverters |
| Approval Number | Wits HREC Reference Number R14/49 Protocol Number M190542 |
| Approval Date | 17 July 2019 |
| Age Eligibility | 18 years and older |
| HIV-1 Testing Schedule | One rapid antibody test at PrEP initiation, 1-month visit and every 3 months^a^ |

**B3. Prevention Options for Women Evaluation Research (POWER)**

| Countries | Kenya and South Africa |
| --- | --- |
| Implementing Partner | University of Washington and Wits RHI |
| Title of Protocol | A Cohort for Evaluation of Open-label PrEP Delivery Among Kenyan and South African Women: The POWER Cohort |
| Approval Number | University of Washington Approval # STUDY00000950. Institutional Review Board (IRB) approvals were also obtained from the Kenya Medical Research Institute, Jaramogi Oginga Odinga Teaching and Referral Hospital (JOOTRH) Independent Ethics Review Committee, University of Cape Town and University of Witwatersrand |
| Approval Date | 30 March 2017 |
| Age Eligibility | Ages 16-25 in Kisumu and Cape Town, ages 18-25 in Johannesburg |
| HIV-1 Testing Schedule | One rapid antibody test at PrEP initiation, 1-month visit and every 3 months ^a,b^  All potential seroconversions were confirmed using HIV EIA |

^a^ Algorithm in South African National HTS Guidelines was followed

^b^ Algorithm in Kenya HTS Guidelines was followed
